# MOUSE SKELETAL MUSCLE OVEREXPRESSION OF TFEB: AN EXERCISE MIMETIC PROMOTING BRAIN HEALTH INDEPENDENT OF AHN

**DOI:** 10.1093/geroni/igae098.3676

**Published:** 2024-12-31

**Authors:** Mia Hakian, Ian Matthews, Catherine Cai, Constanza Cortes

**Affiliations:** University of Southern California, Los Angeles, California, United States; University of Southern California, Los Angeles, California, United States; University of Southern California, Los Angeles, California, United States; University of Southern California, Cortes Lab, Los Angeles, California, United States

## Abstract

Adult hippocampal neurogenesis (AHN), which forms new neurons in the dentate gyrus of the hippocampus, is important for cognition and decreases with age. This reduction in AHN can be reversed with voluntary wheel running in mice, increasing the expression of Transcription Factor E-B (TFEB) within skeletal muscle. TFEB regulates lysosomal and metabolic function through the expression of genes controlling mitochondrial oxidation, fatty acid oxidation, and oxidative phosphorylation. Our lab has recently shown that a transgenic mouse model that overexpresses TFEB only in skeletal muscle mimics many of the neuroprotective benefits of exercise during aging compared to the control, but the effect of TFEB on AHN was unknown. Therefore, we hypothesized that overexpression of TFEB in skeletal muscle has the potential to increase AHN in our exercise mimicking TFEB model. Levels of AHN were measured by using immunofluorescence approaches in hemibrain cryosections to quantify the number of doublecortin (DCX) positive cells, a marker for immature neurons, in the dentate gyrus of the hippocampus of 3-month-old control and muscle-TFEB overexpressing mice. If skeletal muscle-TFEB overexpression is an exercise mimetic, then we expect to see increased levels of AHN in the DG of the hippocampus in TFEB mice compared to the control. We found no significant difference of AHN in TFEB mice compared to the control. These results contradict our hypothesis, but are interesting because we show the maintenance of memory function in our old muscle TFEB mice. This suggests our muscle TFEB mice mimic exercise’s improvement of hippocampal function independent of AHN.

